# Spinal Chondrosarcoma: A Review

**DOI:** 10.1155/2011/378957

**Published:** 2011-03-08

**Authors:** Pavlos Katonis, Kalliopi Alpantaki, Konstantinos Michail, Stratos Lianoudakis, Zaharias Christoforakis, George Tzanakakis, Apostolos Karantanas

**Affiliations:** ^1^University Hospital, University of Crete, Heraklion 711 10, Greece; ^2^Department of Histology, Medical School, University of Crete, Heraklion 710 03, Greece; ^3^Department of Radiology, University Hospital, University of Crete, Heraklion 711 10, Greece

## Abstract

Chondrosarcoma is the third most common primary malignant bone tumor. Yet the spine represents the primary location in only 2% to 12% of these tumors. Almost all patients present with pain and a palpable mass. About 50% of patients present with neurologic symptoms. Chemotherapy and radiotherapy are generally unsuccessful while surgical resection is the treatment of choice. Early diagnosis and careful surgical staging are important to achieve adequate management. This paper provides an overview of the histopathological classification, clinical presentation, and diagnostic procedures regarding spinal chondrosarcoma. We highlight specific treatment modalities and discuss which is truly the most suitable approach for these tumors. Abstracts and original articles in English investigating these tumors were searched and analyzed with the use of the PubMed and Scopus databases with “chondrosarcoma and spine” as keywords.

## 1. Introduction

According to the World Health Organization, chondrosarcomas represent a heterogenous group of tumors characterized by their ability of cartilage formation [1]. Chondrosarcoma is the third most common primary malignant bone tumor after osteosarcoma and Ewing's sarcoma. However, the incidence of spinal chondrosarcomas is estimated to be from 2% to 12% in various series [2]. The thoracic spine is the most frequent localization, followed by the cervical and lumbar region [3]. Unlike most other malignant spinal tumors, the lesions may arise in the vertebral body (5%), the posterior elements (40%), or both (45%), since there are three growth centers in each vertebra from which the tumor originates [4]. The most common presenting symptom in chondrosarcoma is pain. Other complaints include a palpable mass and neurologic deficits in half of the patients [3].

 The radiological features of chondrosarcomas vary significantly depending upon the histologic grade. The spectrum of findings starts with lysis, which is difficult to discriminate form enchondromas. High-grade tumors are demonstrated radiographically with a moth-eaten destruction and interrupted periosteal reaction. Higher grade of differentiation is related to the presence of a “rings and arcs” pattern of calcification into the tumor matrix. The differential diagnosis depends on the presence of calcifications. If present, then the main consideration is enchondroma. If absent, many lesions should be also considered such as metastases, malignant fibrous histiocytoma, and fibrosarcoma. The following criteria favor a diagnosis of chondrosarcoma: deep endosteal scalloping (>2/3 of cortical thickness), cortical disruption, periosteal reaction, soft tissue mass, and intense radionuclide uptake. Associated soft tissue mass is a common finding, and, thus, CT or MRI are important to fully appreciate the extraosseous extension [5].

The histologic grading is just one indicator that can predict the tumor's biological behavior. Prognosis is also related to management. The clinical challenge is to prevent recurrence and to optimize treatment options. Chondrosarcomas are typically resistant to known protocols of radiotherapy and chemotherapy; therefore, surgical removal is essential, and the outcome is based on the margins achieved [6, 7]. This review focuses on the most relevant issues relating to classification, diagnostic work-up, and surgical management of spinal chondrosarcomas. The principles of surgical excision and reconstruction as well as novel treatment options like radiofrequency ablation and cryosurgery are also discussed.

## 2. Histopathological Classification of Chondrosarcoma

Chondrosarcoma has been classified into conventional and variant types. The variant types of chondrosarcoma include the least aggressive *clear cell* type and the *high-grade* mesenchymal and dedifferentiated tumors associated with poor prognosis. Conventional chondrosarcoma, which constitutes approximately 85% of all chondrosarcomas, is further classified into *primary *(85%) and *secondary* (15%) [8]. The primary chondrosarcoma arises *de novo* within the bone and can extend through the cortex with a large soft-tissue mass. A secondary chondrosarcoma develops on the surface of the bone mostly as a result of malignant transformation within the cartilage cap of a pre-existing benign tumor such as osteochondroma [1, 9]. It has been reported that secondary chondrosarcomas tend to be of a lower grade exhibiting a better prognosis than primary tumors [10]. In general, primary and secondary chondrosarcomas are histologically similar, and, for both, three different grades are recognized, which is one of the most reliable predictors of clinical behavior [11]. These histological grades are directly connected with prognosis and the risk of metastases. Grade I tumors are characterized by low cellularity and lack of pleomorphism; they contain a rich hyaline cartilage matrix and rarely metastasize [12]. In contrast, grade III chondrosarcomas are extremely cellular with pleomorphism and mitotic figures. Mucomyxoid matrix areas are frequent in grade III tumors and metastases occur in 70% of patients. Grade II chondrosarcoma hold some of the characteristics of both grade I and grade III [11]. In addition, to histological grade of the lesion, the prognosis depends on the possibility of performing *en bloc *excision with proper oncologic margins. Because of the difficulties associated with *en bloc *surgery in the spine, tumors of the vertebral column have had a deprived prognosis independent of the histological grade [9]. 

 It seems that chondrosarcomas may be biologically dynamic, since up to 13% of recurrent tumors display a higher grade of malignancy or even dedifferentiation compared to the initial neoplasm, with a severe adverse prognosis. Alterations in TP53 as well as the *CDKN2A *(p16) tumor suppressor gene are thought to be important for the progression of low-grade towards high-grade chondrosarcoma. [13, 14].

Although primary and secondary chondrosarcomas show similarity in histopathologic features, they differ at the molecular genetic level [14]. The exostosin (*EXT*) genes, which are connected with the development of multiple osteochondromas (MOs), are involved in the origin of osteochondroma and secondary chondrosarcoma. The *EXT* genes participate in heparan sulphate biosynthesis and the resulting heparan sulphate proteoglycans (HSPGs) are fundamental for cell signaling [15]. Although it is quite clear that inactivation of EXT1 and EXT2 encourages osteochondroma development, the exact molecular trigger causing its malignant transformation is unclear [16]. It is evident that several growth-signaling pathways which are normally activated during skeletal growth such as the Indian hedgehog (IHH)/parathyroid hormone-like hormone (PTHLH) factor, wingless type (Wnt) protein, and transforming growth factor (TGF) signaling pathways are deprived in secondary chondrosarcoma. The IHH signaling and the Wnt signaling are downregulated while the TGF signaling and the PTHLH signaling, which is downstream of the IHH and it is responsible for chondrocyte proliferation, are up regulated and increased with increasing histological grade [13, 16]. 

On the contrary, *EXT* genes are not involved in the development of primary chondrosarcoma, and, in this case, the initiate event remains unidentified [17]. These tumors are usually aneuploid, with complex karyotypes, and 96% of them contain alterations at some level in the pRb pathway [18].

## 3. Rare Chondrosarcoma Subtypes

In addition to conventional chondrosarcoma, several variant subtypes of chondrosarcoma are recognized which are extremely rare especially when they originate in the spine [19]. 

*Clear cell chondrosarcoma *is a rare variant chondrosarcoma with relatively good prognosis. It is described as a “round cell” neoplasm with clear, empty cytoplasms. Benign giant cells may be present, which is the reason that it might erroneously be diagnosed as a chondroblastoma. Vascularity is a common feature in this tumor. Although it has a reasonably benign biological behavior, clear cell chondrosarcoma needs to be treated as a malignancy. Metastases are rare, but may occur up to 20 years following initial diagnosis; consequently, long-term followup is required [20]. On the molecular level, recent studies have shown that there is evidence of extra copies of chromosome 20 and loss or rearrangements of 9p. Also, expression of PTHLH, PDGFIHH, Runt-related transcription factor 2, and matrix metalloproteinase 2 [21, 22] were found. 

*Mesenchymal chondrosarcoma* is another rare variant of chondrosarcoma, which is highly malignant. The prognosis of this tumor is extremely poor. It can involve both the bone and soft tissues. Huvos et al. classified mesenchymal chondrosarcoma into hemangiopericytoma-like and small dark round cell type. The same team reported that this tumor occurs in relatively young patients (mean age of presentation 26 years) [23]. Histopathologically, it is characterized by varying amounts of differentiated cartilage admixed with undifferentiated petite round cells [24]. On the molecular level, more than 60% of the tumors demonstrate p53 overexpression. In addition, expression of the antiapoptotic BCL2, protein kinase C- (PKC-), and platelet derived growth factor receptor- (PDGFR-)pathways were found [25, 26].

*Dedifferentiated chondrosarcoma *is an extremely aggressive variant type of chondrosarcoma with deprived prognosis. It is defined as a borderline low-grade chondrosarcoma next to high-grade noncartilaginous anaplastic sarcoma, with a remarkably sharp junction between the two components [27, 28]. These two components hold identical genetic aberrations with additional genetic changes in the anaplastic component, suggesting a common ancestor cell with early diversion of the two components [29].

## 4. Risk Factors and Epidemiology

Several hypotheses have been proposed regarding the risk factors of spinal chondrosarcomas. Moreover, recurrence of chondrosarcoma of the spine is very common in case of invasion of the epidural space [30, 31]. Hereditary multiple exostoses is a syndrome that seems to be connected with spinal chondrosarcoma and constitute a significant risk factor [23]. Furthermore, there are benign lesions, such as chondromas, that can undergo a malignant transformation to spinal chondrosarcoma [22]. Epidemiological data shows a fairly equal gender representation between men and women, a range of age from 13 to 78 years, and a mean age of 33 years [7, 9, 32]. Location of chondrosarcoma involves the lumbar spine in 68% of the cases, the thoracic spine in 23%, and the cervical spine in 9%, and classification as peripheral and central chondrosarcoma is, almost in 2/3 of the cases, in favor of the peripheral [9]. Other studies show that these tumors have higher frequency in the thoracic than the rest of the spine as a result of the greater number of thoracic segments relative to cervical and lumbar regions [7]. Finally, almost 90% of tumors were classified as low grade (Enneking Stage I) and had a greater incidence among Caucasians [7, 9, 32].

## 5. Radiologic Features and Imaging

**Figure 1:**
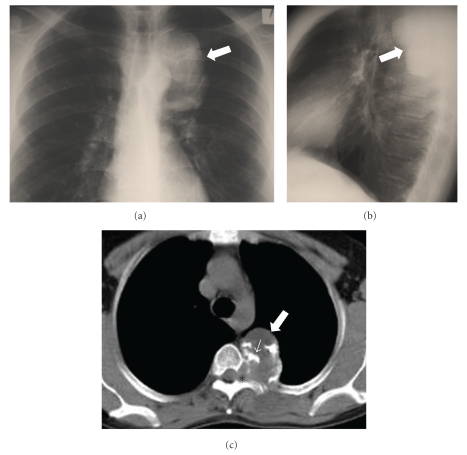
A 32-year-old man with chondrosarcoma. The posteroanterior (a) and lateral (b) chest radiographs, show a well-defined radiopaque lesion in the left posterior paraspinal location (arrows). (c) The axial MDCT image demonstrates a soft-tissue mass (arrow) with amorphous “rings and arcs” calcified matrix (thin arrow) and adjacent neural foramina widening (asterisk).

**Figure 2:**
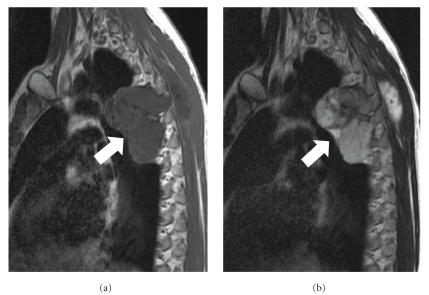
MR imaging of the same patient. The sagittal T1-w (a) MR image shows a hypointense lobulated lesion (arrow). (b) The sagittal T2-w MR image shows the lesion with heterogeneous but predominantly high signal intensity (arrow). Note the superficial palpable component of the tumor (asterisks).

**Figure 3:**
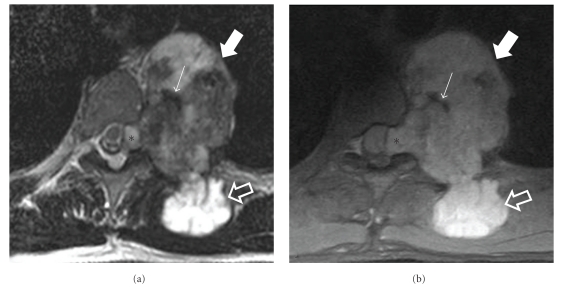
MR imaging of the same patient. The axial T2-w (a) and the axial fat-saturated PD-w (b) MR images show a heterogeneous high intensity mass (thick arrows) with mineralized elements that demonstrate low signal intensity (thin arrow). Note the superficial (open arrows) as well as the neural foraminal extension (asterisks) of the tumor.

**Figure 4:**
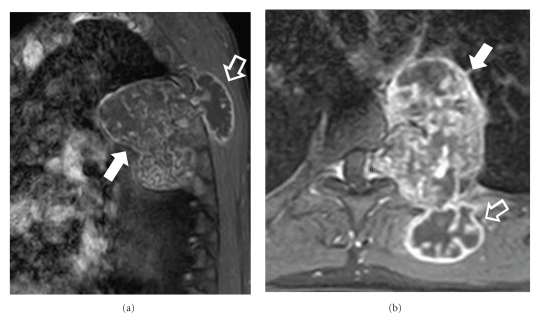
MR imaging of the same patient. The sagittal (a) and axial (b) contrast-enhanced fat-saturated T1-w MR images show intense heterogeneous enhancement of both the intrathoracic (arrows) and the superficial (open arrows) tumor components. Enhancement is also observed in the intraforaminal component of the tumor (asterisk).

**Figure 5:**
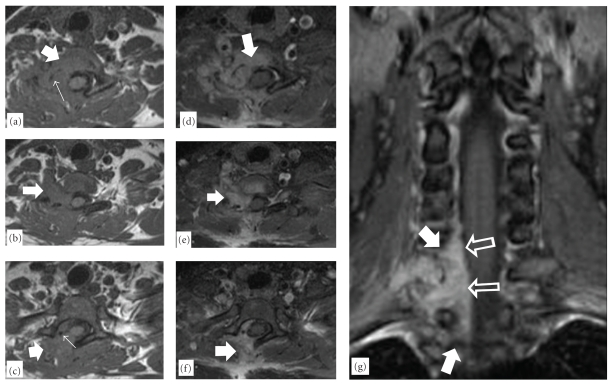
A 41-year-old female with a recurrent chondrosarcoma of the lower cervical spine. The axial T1-w MR images ((a)–(c)), show a soft-tissue mass in the right lower cervical spine (arrows) with foci of calcifications (thin arrows). The fat-suppressed contrast-enhanced MR images ((d)–(f)) show the intense enhancement of the lesion (arrows). (g) The coronal fat-suppressed contrast-enhanced MR image, shows the extension of the lesion within the right epidural space (arrows), with spinal cord displacement (open arrows).

Plain radiographs demonstrate spinal chondrosarcoma as a well-defined mass with internal calcification [33]. In case that the mass projects into the lung fields, a well-defined opacity may be seen (Figures 1(a) and 1(b)). Computed tomography (CT), with its ability to overcome overlying structures, is able to depict the anatomic origin of the lesion and the pattern of calcification, namely, “rings and arcs” (Figure 1(c)). CT may also reveal paravertebral extension of the tumor, the displacement and potential infiltration of the surrounding structures, and involvement of adjacent levels [33–36]. Occasionally, spinal chondrosarcoma may appear as a lytic lesion involving the vertebral body, which may be complicated by a compression fracture of the superior or inferior end-plates [34, 35]. Magnetic resonance imaging (MRI) demonstrates the tumor as a low-signal intensity on T1-w and heterogeneous low and high-signal intensities on T2-w and STIR images, suggesting mineralized and nonmineralized matrices (Figures 2, 3, and 5) [33, 35]. In addition, MRI is better compared to CT in depicting the epidural and intraforaminal extension highlighting possible compression of the neural structures [34]. Fat-suppressed contrast-enhanced T1-w images show peripheral and lobulated rim enhancement (Figure 4) whereas lesions with limited calcification may appear with homogenous enhancement (Figure 5) [33, 35]. Scintigraphy by means of Tc-99m HMDP will show focal accumulation in the tumor site [33].

## 6. Histological Diagnosis and Staging

The histological examination of the spinal chondrosarcoma shows vacuolated tumor cells with irregular hyperchromatic nuclei and clear cytoplasm, encircled by a network of fine osteoid trabeculae and spicules of nontumoral infiltrated bone [33]. In other cases, the tumor manifests a biphasic pattern with solid and cellular proliferation of small round-short spindle tumor cells and differentiated chondroid islands with endochondral ossification [33]. According to Enneking staging system, the lesions are classified as follows: histologically low-grade intracompartmental (IA), histologically high-grade intracompartmental (IIA), histologically low-grade extracompartmental (IB), and histologically high-grade extracompartmental (IIB) (Table 1) [9, 37, 38]. The second column of Table 1 is explained below.

**Grade**

In the Enneking system, bone tumors are graded as follows:

- G0: benign lesion,
- G1: low-grade malignant lesion,
- G2: high-grade malignant lesion.

The third column of Table 1 is explained below.

**Site**

In the Enneking system, the site and local extent of bone tumors are classified as follows:

- T0: a benign tumor that is confined within a true capsule and the lesion's anatomic compartment of origin (i.e., a benign intracapsular, intracompartmental lesion),
- T1: intracompartmental lesion,
- T2: extracompartmental lesion.

The fourth column of Table 1 is explained: metastatic classification in the Enneking system is as follows.

- M0: no regional or distant metastasis,
- M1: regional or distant metastasis.

**Staging**

1. Under the Enneking system, malignant tumors are classified into stages I–III, with further subdivisions into A and B. Grade 1 and grade 2 tumors are stage I and stage II, respectively. T1 and T2 tumors are stage A and stage B, respectively. Tumors with distant metastasis are stage III.

**Figure 6:**
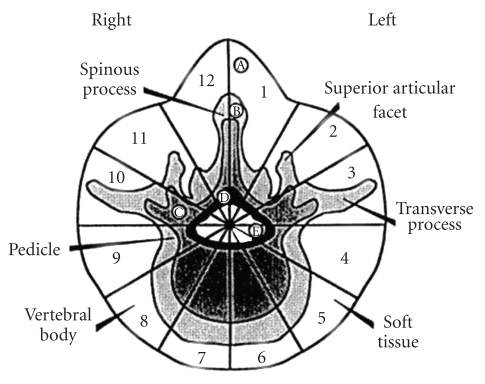
Weinstein-Boriani-Biagini surgical staging for spinal tumors, the transverse plane, and into five layers (A to E, from the paravertebral extraosseous region to the dural involvement). [40]. (A) Extraosseous (soft tissues), (B) intraosseous (superficial), (C) intraosseous (deep), (D) extraosseous (extradural), (E) extraosseous (intradural), and (M) metastasis.

Furthermore, the extent of the lesions has been classified according to the Weinstein-Boriani-Biagini (WBB) staging system with data taken from radiographs, CT and MRI scans, and surgical reports (Figure 6) [9, 39]. The vertebral body is topographically divided in twelve zones similar to the clock hours and five layers beginning from the paravertebral bony compartment until the meningeal layer, and the site of the tumor is recorded. Finally, the Tomita staging is as follows: lesion within the vertebral body (I), the lesion extends to the pedicle (II), lesion extends to the whole vertebra (III), extension to epidural space (IV), extension to paravertebral space (V), extension to paravertebral space and neighboring vertebral levels (VI), and extension to multiple levels (VII) [9, 30, 33]. 

Even though primary spinal chondrosarcoma is uncommon, it represent an enormous therapeutic challenge. Consequently, it is necessary a reliable, validated, and evidence-based classification system on which to base treatment and conduct future research. A resent study shows that the intraobserver reliability for both Enneking and WBB classifications are substantial to near perfect; however, the interobserver reliability was considered fair to moderate. Therefore further work is needed to investigate the validity of these classification systems [40].

## 7. Differential Diagnosis

 Tumors to be included in the differential diagnosis are the angioblastic meningioma, osteosarcoma, Ewing's sarcoma, and hemangiopericytoma. However, their histological features are distinct [36]. In case of a coexistence of a pathologic fracture, osteoporosis should be excluded from the diagnosis [35].

## 8. Management and Outcome (Prognosis)

The inherent resistance of chondrosarcoma to conventional radiation and chemotherapy makes the choice of surgical resection an inevitable necessity for patients suffering from such a tumor [9]. A proper oncologic [37, 38] (Enneking) and surgical [31, 39] (Weinstein- Boriani- Biagini) staging of the tumor by a multidisciplinary oncologic team is a prerequisite for making the right decision on the most appropriate surgical technique, combined or not with any adjuvant medical modalities [31, 41]. Once a biopsy is to be undertaken before the definite procedure, this should be part of the whole treatment plan and carried out under the guidance and supervision of the spine surgeon [19, 41]. A closed CT-guided biopsy using a 16–18G trocar instead of a fine needle is preferred from an open one [19, 41], as being the most correct according to the oncologic rules and principles. It is fundamental that the biopsy path is contained by the excision margins at the definite surgery [19, 41]. 

### 8.1. Surgery

Surgery is of critical importance when treating spinal chondrosarcomas and should aim at preserving or even improving functionality, relieving pain, and controlling local tumor recurrence, promising a prolonged survival [31]. The spectrum of oncologically established surgical procedures applicable to the spinal column [39] varies from the most complex *en block* resection (defined as an attempt for surgical tumor removal in a single piece surrounded by healthy tissue) to the simplest one implying a piecemeal removal of the tumor (curettage). *En block* resection should be accompanied by a histological inspection and description of the resected margins [39], defined as “wide” (through the healthy tissue outside the pseudocapsule [37–39]. 

*En block* resection for primary treatment of chondrosarcoma successfully performed, wide, with disease-free margins, provides the best results regarding local tumor control with reported rates of recurrence as low as 3–8% [9, 42]. In contrast, an intralesional or curettage procedure is deemed to be unacceptable with regression rates up to 100% [3, 5, 9]. Recurrence usually appears within 3–5 years postoperatively [7, 9, 30] and much closer to the operation when a subtotal excision instead of an *en block* resection had been performed [7, 9]. However, isolated cases of late relapse as far as 10 years have been described making a long-term follow-up period for these patients essential [9]. 

Similarly, *en block *excision with negative histological margins offers patients the greatest chance for a prolonged survival compared with any other procedure resulting in subtotal resection, and tumor-related death estimated 12% versus 42%, respectively, for the two groups during the follow-up period [7, 9, 19, 31, 42]. Local recurrences after intralesional debulking procedures can be treated with operations of adequate margins and may give satisfactory results whereas a repetition of curettage does not prevent recurring even if accompanied by radiation [9, 19, 30]. 

Although *en block* resection with tumor-free margins is the optimum surgical treatment for spinal chondrosarcomas that guarantees a long recurrence-free interval and patient survival [3, 5, 7, 9, 19], at the same time, induces a significant surgical challenge [31, 41], quite often requiring a spondylectomy. The proximity of neural, vascular, and visceral structures combined with the complex spinal anatomy makes the goal of wide margins a difficult task, which is not always feasible even if a meticulous preoperative plan has been employed [3, 5, 9, 41, 43]. Complications deriving from *en block* excision are not meaningless, comprising mainly of wound problems and blood loss for the early postoperative period and implant failure and local regression for the late period [3, 41, 43]. 

In recent years, the innovative work of WBB [39] and Tomita [44] on surgical staging of spine tumors in combination with the evolution of modern surgical techniques [32, 33, 43, 45–47] regarding approach, reconstruction, and stabilization of vertebral column without endangering nerve structures and functional outcome or compromising the oncologic result have demonstrated that *en block* primary spine tumor resection, like chondrosarcoma, may be a feasible and oncologically justified procedure [40], provided that an experienced oncological multidisciplinary team has set the indication and properly planned it [41, 42].

### 8.2. Radiation and Chemotherapy

Both radiation and chemotherapy have been used as adjuvant therapies after completion of surgery [5, 7, 9], but their positive effect on patient survival and local tumor recurrence seems to be of little importance [3, 5, 7, 9, 43]. Chemotherapeutic agents have not proved to affect the outcome at all in spinal chondrosarcoma and their role is limited [5, 7, 9].

A reasonable explanation of chemotherapy incompetence might be expression of the multidrug-resistance 1 gene, P glycoprotein, resulting in resistance to doxorubicin in vitro. Also, the large amount of extracellular matrices, the poor vascularity, and the low proliferation rate of the tumor cells make chemotherapy agents even more ineffective [13]. Tumors with small cells and low percentage of cartilage matrix show more sensitivity to chemotherapy. Mesenchymal chondrosarcoma, although there is lack of prospective studies, seems to be responsive to doxorubicin-based combination chemotherapy. These patients should be considered for adjuvant chemotherapy, and in the case of metastatic disease, palliative chemotherapy [23]. Yet there is a pressing need for new standard chemotherapy treatment options for the patients with unresectable or metastatic disease. Recently, new chemotherapy agents such as histone deacetylase and aromatase inhibitors as well as angiogenesis inhibitors have been studied in vitro and in vivo, and several studies are currently ongoing [13]. 

 Although radiotherapy is frequently used in patients with inadequate margins [5], survival for these patients remains low compared to those who had a margin-free *en block* excision and no adjuvant radiation [9, 43]. One reason explaining these results, apart from tumor resistance, could be the fact that these modalities are implemented mainly on chondrosarcomas of higher grade or patients who cannot tolerate a major operation [7]. Radiotherapy applied in high doses (65 Gy) [48] or proton beam radiation [49, 50] becomes mostly important when treating chondrosarcoma of the upper cervical spine, due to the technical difficulties that an effort for wide surgical excision in this peculiar anatomical location entails. Local control rates of up to 92% have been reported [50] but the follow-up period is short (<5 years). Recently, a systematic review [42], including a multicenter cohort, concludes that radiation as an adjunct to surgery, in case that an incomplete excision of the mass has been achieved, may have a small beneficial effect on outcome and mainly on local tumor control. Radiation as a primary treatment for chondrosarcoma of the spine is strongly not indicated [42].

### 8.3. Cryosurgery and Radiofrequency Ablation

Although latest publications report the effectiveness of cryoablation in combination with curettage, as an alternative to more radical procedures, for the treatment of low grade I chondrosarcoma of the appendicular skeleton [51, 52], this is not documented by the current literature regarding the mobile spine. Radiofrequency ablation is another minimal invasive percutaneous technique used mainly for palliating painful skeletal metastasis [53–55], including the spine region [56], but there is no study, to our knowledge, addressing the application of this technique in primary spinal tumors and more specifically chondrosarcoma.

### 8.4. Prognosis

Besides, histological grade of the tumor, the prognosis depends on the possibility of performing *en bloc *excision with appropriate oncologic margins. A successful operation, in terms of complete tumor excision with disease-free margins is a major independent prognostic factor for a favorable course of the disease, affecting critically both local tumor control and patient survival [5, 7, 9, 19, 42]. Regarding tumor recurrence, it is reported to rate higher (up to 100%) when inadequate margins (intralesional or contaminated) have been accomplished during the operation [7, 9, 19, 41, 42] and/or a primary treatment (including biopsy) has taken place outside the reference center [19, 41]. Distal metastases are sparsely reported in the literature [7, 41], occurring during the course of the disease and related to a higher tumor grade [41] and a local tumor recurrence [19].

Regarding survival, it is difficult to extract accurate rates due to the lack of large series and standardization of surgical techniques in the existing literature. However, York et al. [7] estimate an overall 5- and 10-year survival rate at 64% and 40%, respectively, for 21 surgically treated patients. Similarly, Bergh et al. [19] in their study of 69 cases of the axial skeleton (including 12 spinal chondrosarcomas) calculate overall 5-, 10-, and 15-year survivals for the whole series at 72%, 67%, and 63%. Factors adversely affecting survival are considered an older patient age and a higher tumor grade [19], inadequate surgical margins [5, 19, 41], and a local recurrence [9, 19, 42]. Failed local control of the disease, as a consequence of insufficient surgery, is deemed to be crucial for survival, with a rate of tumor-related death as high as 61% for patients suffering a local regression [19].

## Figures and Tables

**Table 1:** The Enneking system for the surgical staging of bone and soft-tissue tumors is based on grade (G), site (T), and metastasis (M) [40].

Stage	Grade	Site	Metastasis
IA	G1	T1	M0
IB	G1	T2	M0
IIA	G2	T1	M0
IIB	G2	T2	M0
III	G1 or G2	T1 or T2	M1
